# Evaluation of a point-of-care haemozoin assay (Gazelle device) for rapid detection of *Plasmodium knowlesi* malaria

**DOI:** 10.1038/s41598-023-31839-7

**Published:** 2023-03-23

**Authors:** Angelica F. Tan, Priyaleela Thota, Sitti Saimah Binti Sakam, Yao Long Lew, Giri S. Rajahram, Timothy William, Bridget E. Barber, Steven Kho, Nicholas M. Anstey, David Bell, Matthew J. Grigg

**Affiliations:** 1 Global and Tropical Health Division, Menzies School of Health Research, Charles Darwin University, Darwin, PO Box 41096, Casuarina, NT 0810 Australia; 2 Infectious Diseases Society Kota Kinabalu Sabah–Menzies School of Health Research Clinical Research Unit, Kota Kinabalu, Sabah Malaysia; 3 Hemex Health, Portland, OR USA; 4 Hospital Queen Elizabeth II, Kota Kinabalu, Sabah Malaysia; 5 Clinical Research Centre, Queen Elizabeth Hospital, Ministry of Health, Kota Kinabalu, Sabah Malaysia; 6 QIMR Berghofer Medical Research Institute, Brisbane, Australia

**Keywords:** Malaria, Diagnosis

## Abstract

*Plasmodium knowlesi* is the major cause of zoonotic malaria in Southeast Asia. Rapid and accurate diagnosis enables effective clinical management. A novel malaria diagnostic tool, Gazelle (Hemex Health, USA) detects haemozoin, a by-product of haem metabolism found in all *Plasmodium* infections. A pilot phase refined the Gazelle haemozoin identification algorithm, with the algorithm then tested against reference PCR in a larger cohort of patients with *P. knowlesi* mono-infections and febrile malaria-negative controls. Limit-of-detection analysis was conducted on a subset of *P. knowlesi* samples serially diluted with non-infected whole blood. The pilot phase of 40 *P. knowlesi* samples demonstrated 92.5% test sensitivity. *P. knowlesi*-infected patients (*n* = 203) and febrile controls (*n* = 44) were subsequently enrolled. Sensitivity and specificity of the Gazelle against reference PCR were 94.6% (95% CI 90.5–97.3%) and 100% (95% CI 92.0–100%) respectively. Positive and negative predictive values were 100% and 98.8%, respectively. In those tested before antimalarial treatment (*n* = 143), test sensitivity was 96.5% (95% CI 92.0–98.9%). Sensitivity for samples with ≤ 200 parasites/µL (*n* = 26) was 84.6% (95% CI 65.1–95.6%), with the lowest parasitaemia detected at 18/µL. Limit-of-detection (*n* = 20) was 33 parasites/µL (95% CI 16–65%). The Gazelle device has the potential for rapid, sensitive detection of *P. knowlesi* infections in endemic areas.

## Introduction

The public health implications of emerging *Plasmodium knowlesi* infections across most Southeast Asia countries^1–3^ is exemplified by the significant ongoing burden of zoonotic malaria in Malaysia, with 17,125 cases and 48 deaths since 2017^4^ despite near-elimination of other human *Plasmodium* species^5,6^. Rapid and accurate detection remains a crucial pillar of effective clinical management for zoonotic and human-only malaria as recommended by the WHO in all endemic settings^7^. Delays in treatment for *P. knowlesi* infections can lead to the development of severe disease and increased risk of fatal complications^8^.

Microscopy is the current standard for malaria point-of-care (POC) diagnosis in most endemic countries in Southeast Asia but is unreliable for *P. knowlesi* due to misidentification with other *Plasmodium* species^9,10^. Microscopy also requires trained laboratory technicians and equipment, necessitating ongoing quality assurance^11^. In Malaysia, the implementation of routine polymerase chain reaction (PCR) in *Plasmodium* species confirmation for all malaria cases since 2012^12^ has enabled accurate public health reporting for *P. knowlesi*^13^. Although demonstrating an increasing *P. knowlesi* case incidence, particularly in East Malaysia^14^, routine PCR is not feasible for immediate clinical management and requires expensive laboratory infrastructure and trained personnel.

The World Health Organization (WHO) Evidence Review group first recommended developing and improving POC *P. knowlesi* detection methods in 2017 in response to the increasing public health threat posed by zoonotic malaria transmission with an intractable monkey parasite reservoir^15^. One novel diagnostic approach utilizes rotating magneto-optical technology for the detection of haemozoin crystals, a paramagnetic by-product found in all malaria species^16^. The Gazelle™ (Hemex Health, USA) is a battery-operated device. It functions by detecting changes in light signal intensity as it passes through magnetically-induced reorientated haemozoin crystals found in *Plasmodium*-infected blood samples collected from a finger prick or venous sampling. Previous studies have demonstrated the efficacy of the Gazelle™ in detecting *P. falciparum*^17^ and *P. vivax*^18^ when compared to nested PCR as the reference standard^19^. In addition to reporting detection limits for *Plasmodium* species infections as low as 40 parasites per microliter of blood^20^, the Gazelle™ was able to detect haemozoin in malaria-infected whole blood across early and late asexual stages of the parasite life-cycle^21^. Existing haemozoin-based detection assays have not been designed or evaluated for *P. knowlesi* human infections.

In this study, the performance of the Gazelle™ in detecting clinical *P. knowlesi* infections compared to reference PCR was assessed in a setting endemic for *P. knowlesi*.

## Methods

### Study site and subjects

**Figure 1:**
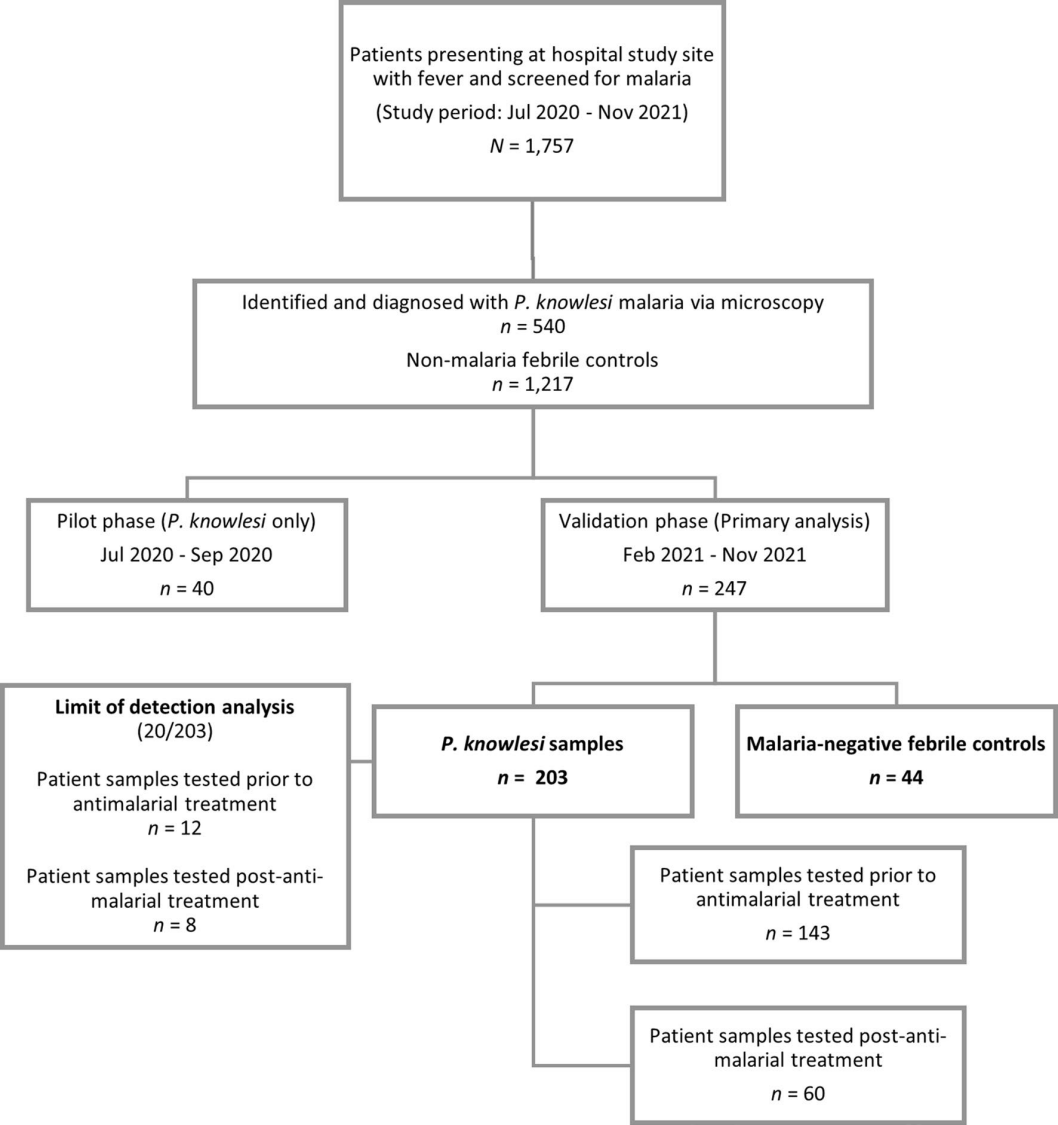
Study enrolment flow chart and number of samples tested.

Malaria patients were enrolled between July 2020 and November 2021 at Ranau District Hospital in Sabah, Malaysia (Fig. 1). Ranau District Hospital is a secondary referral center servicing the administrative district (area of 3609 km^2^), including 16 primary health clinics, with all cases of malaria admitted for in-patient management according to national guidelines^12^. Patients presenting at the hospital study site were included if they were positive for *P. knowlesi* by microscopy, were aged > 1 year, and they or their guardian provided appropriate written informed consent. Appropriate written informed consent was obtained from febrile individuals aged more than 12 years with negative malaria microscopy at the same healthcare facility, prospectively enrolled as controls. Patient blood samples were collected, and their demographic and clinical information were recorded on standardized case record forms as part of an ongoing prospective observational malaria study. Severe knowlesi was defined using WHO 2014 research criteria^22^ for *P. knowlesi*, including hyperparasitaemia threshold of 100,000/μL, and jaundice defined as bilirubin > 50 μmol/L with parasite count > 20,000/μL and/or creatinine > 132 μmol/L^23,24^.

### Ethical approval

The study was approved by the Medical Research and Ethics Committee of the Ministry of Health, Malaysia (NMRR-10-584-6684) and Menzies School of Health Research, Northern Territory, Australia (HREC 10-1431). Written informed consent was obtained from all participants and/or their legal guardians. All methods were performed in accordance with relevant guidelines and regulations.

### Blood sample procedures

Venous whole blood samples were collected in ethylenediaminetetraacetic acid (EDTA) vacutainers prior to antimalarial treatment where possible. Thick and thin blood films were generated to verify parasite count by microscopy. Microscopic quantification of *P. knowlesi* parasitaemia was conducted by an experienced research microscopist equivalent to WHO Level 1 competency. The final parasite count per microlitre was calculated from the number of parasites counted to 200 white blood cells on a thick blood film, multiplied by the individual patient’s total white cell count^25^ obtained from routine hospital laboratory automated counts. The percentage of each parasite developmental stage^26^ was determined based on the number of early rings, late rings, mature trophozoites, schizonts, and gametocytes in 40 high-powered fields (HPF, × 1000 magnification) on thin blood films.

A 30 µL blood sample (as specified in the manufacturers’ instructions) was used to evaluate the Gazelle™^17,27^. The remaining volume of EDTA whole blood samples were stored at − 80 °C for subsequent malaria PCR species confirmation.

### *Plasmodium* species confirmation by real-time PCR

Malaria PCR was conducted on all microscopically diagnosed malaria cases and microscopy-negative controls for the following *Plasmodium* species: *P. knowlesi, P. falciparum, P. vivax, P. malariae* and *P. ovale* spp. Genomic DNA was extracted from 200 μL of whole blood using QIAamp DNA Blood Mini Kits (Cat. No.: 51106; QIAGEN) according to the manufacturer’s manual, with a final elution volume of 200 μL. PCR was performed by laboratory research members blinded to the microscopy results. A real-time PCR assay, QuantiFast™ targeting the 18S SSU rRNA gene was conducted once for each sample as per the manufacturer’s and Sabah Public Health Laboratory protocols, using the Bio-Rad CFX96 Touch™ PCR machine (Bio-Rad, USA). QuantiFast™ real-time PCR was carried out over two separate reactions. The first was for *Plasmodium* genus screening, and if positive, was followed by subsequent *P. knowlesi* and other human *Plasmodium* species-specific detection^28^.

### Study procedures

An initial pilot phase was conducted to refine the Gazelle™ detection algorithm for *P. knowlesi*. Whole blood samples of microscopy-identified positive *P. knowlesi*, which were later confirmed via PCR, were placed into separate test cartridges, consisting of 30 µL of whole blood sample and 65 µL of assay buffer, then loaded into the Gazelle™ device^17,27^. Test results were displayed on the device screen within a minute and systematically documented by a trained research staff member. Each sample was tested only once. In the event of an invalid test result, a new cartridge was prepared to repeat the test. Daily calibration of the Gazelle™ device was conducted prior to testing of clinical samples and comprised running a haemozoin positive control (active ingredients: Triton X-100 [assay buffer], propylene glycol, FD&C Red 40, citric acid, sodium benzoate [food dye] and concentrated artificial haemozoin solution), negative control (ingredients: assay buffer and dye), and an empty cartridge.

Pilot data from the Gazelle™ were uploaded to Hemex servers in real-time. Algorithm refinement was conducted at the completion of this initial study phase by reducing background noise, updating algorithm threshold values used for positive or negative detection, and optimizing the speciation algorithm for *P. knowlesi* against *P. falciparum*.

The refined detection algorithm was then tested on a subsequent larger prospective cohort of patients diagnosed with *P. knowlesi* malaria as well as malaria-negative febrile controls. A single empty cartridge was run daily as a blank sample negative control prior to testing clinical samples. Similar to the pilot phase, all patient samples were tested only once on the device.

The secondary study analysis evaluated the *P. knowlesi* parasite density limit of detection for the Gazelle™ device. Whole blood samples in EDTA from *P. knowlesi* infected patients with research microscopy parasite quantification were diluted using ABO blood-group matched (Lorne Laboratories Ltd., UK) whole blood from healthy donors at twofold serial dilutions. Sample testing was performed up to the dilution whereby two consecutive negative readings were obtained. The limit of detection was defined as parasite concentration of the dilution tested prior to consecutive negative results. To increase the number of samples in this analysis based on the availability of matched blood group donors, the limit of detection was also tested on a subset of *P. knowlesi* clinical samples taken from patients who had already received anti-malarial treatment. To confirm and improve the quality and reliability of the limit of detection findings, research microscopy readings were also carried out on individual samples at each of the LOD dilutions.

### Statistical analysis

The diagnostic accuracy of the Gazelle™ to detect *P. knowlesi* clinical infections for both the pilot phase and primary analysis were compared against reference PCR results using STATA v16 (TX, USA). Wilcoxon rank-sum test compared differences between the sex of patients (% of males) with age (median) as a dependent variable. The distribution of microscopy parasite counts for infections within defined groups were summarised and compared using the geometric mean. As defined below using the number of true positive (TP), false negative (FN), false positive (FP) and true negative (TN) results, diagnostic tests for sensitivity (TP/TP + FN), specificity (TN/TN + FP), and positive and negative predictive values were calculated^29^. Exact binomial confidence intervals of 95% for each of the above diagnostic metrics were calculated and reported. Overall device performance was compared by testing equality of the receiver operating characteristic (ROC) areas.

Independent comparisons between the sensitivity of the Gazelle™ device versus reference PCR were conducted using Fisher’s exact test for: (a) parasitaemia of above or below 200 parasites/µL, and (b) samples collected before versus after administration of antimalarial treatment. For the latter, logistic regression was used to compare the device sensitivity further when controlling for median time in hours post-treatment. Logistic regression was used to confirm log-transformed parasite counts influenced Gazelle™ test positivity. For the limit of detection analysis, geometric mean LOD with 95% confidence intervals were estimated and reported using GraphPad Prism v9.4.1 (San Diego, CA). A p-value of less than 0.05 for 2-sided tests was considered significant. 

## Results

### Study participant screening and enrolment

From July 2020 to November 2021, 1757 febrile patients presented at the hospital study site, including 540 individuals diagnosed with *P. knowlesi* malaria using screening by microscopy. Of this number, 513 (95%) received species confirmation via real-time PCR, as required by the Malaysian Ministry of Health guidelines for malaria.

A total of 287 febrile patients were enrolled into this study (Table 1), with a pilot cohort comprising of 40 *P. knowlesi* cases, and a validation cohort with 203 *P. knowlesi* cases and 44 malaria-negative controls. Overall, 45% (110/243) of *P. knowlesi* cases and 80% (32/44) negative controls presented directly at the hospital and were not referred from catchment area clinics as mandated for all cases of microscopically confirmed malaria. Extrapolating the proportion of PCR-confirmed *P. knowlesi* cases and malaria-negative controls directly presenting at Ranau hospital yielded a crude estimate of background malaria prevalence of 18.8% (95% CI 16.7–21.1%) for this febrile cohort.

**Table 1:** Demographic and clinical data summary for pilot and validation cohorts.

Variable	Pilot	Validation			
	*P. knowlesi* cases	*P. knowlesi* cases	P-value (*P. knowlesi* validation vs pilot)	Febrile controls (malaria-negative)	P-value (*P. knowlesi* validation vs controls)
Number tested	40	203	–	44	–
Median age (Range), years	28 (9–69)	36 (4–87)	0.021	40 (12–80)	0.15
Sex, N (%) males	30 (75%)	162 (80%)	0.59	31 (70%)	0.17
Parasitaemia geometric mean (95% CI), µL	577 (331–1006)	837 (638–1099)	0.27	–	–
Severe malaria, N (%)	0	2 (1%)	0.53	–	–
History of self-reported previous malaria, N (%)	13 (33%)	83 (41%)	0.33	5 (11%)	< 0.001
Enrolled post anti-malarial treatment, N (%)	27 (68%)	60 (30%)	0.35	–	–
Referred from primary health clinic, N (%)	10 (25%)	103 (51%)	0.002	12 (27%)	0.005
Median fever duration, days (Range)	4 (2–8)	4 (1–14)	0.70	4 (2–21)	0.61

### Pilot phase cohort demographics and Gazelle™ performance

The 40 *P. knowlesi-*infected patients in the pilot phase had a geometric mean parasite count of 577/µL (95% CI 331–1006/µL), ranging from 34 to 14,888/µL (Table 1). Patients had a median age of 28 years (range 9–69 years), with males being the majority (75%). There were 27 samples (68%) which were collected after the patient had been administered anti-malarial treatment.

For patients with no prior treatment, the Gazelle™ achieved a sensitivity of 100% (13/13, 95% CI 75.3–100%) for *P. knowlesi* detection when compared to reference PCR, including a sample with low parasite count of 52 parasites/µL. When samples collected from patients who had already received anti-malarial treatment were included, sensitivity decreased to 92.5% (37/40, 95% CI 78.6–97.6%) (Table 2). The median time post-treatment for the pilot phase enrolments was 3.6 h (IQR 2.8–5.3). There were three false negative results, from samples collected at 13.7, 13.3 and 3.2 h after first anti-malarial treatment dose, and with parasite counts of 34/µL, 38/µL and 105/µL, respectively.

**Table 2:** Diagnostic accuracy of the Gazelle device for *P. knowlesi* detection during pilot and validation cohorts.

Anti-malarial status	Pre-treatment	Post-treatment	Combined^†^
Pilot			
Number tested	13	27	40
Sensitivity, *n*/*N*	13/13	24/27	37/40
%	100	88.9	92.5
(95% CI)	(75.3–100)	(69.4–96.6)	(78.6–97.6)
Validation			
Number tested	143	60	203
Sensitivity, *n*/*N*	138/143	54/60	192/203
%	96.5	90.9	94.6
(95% CI)	(92.0–98.9)	(79.5–96.2)	(90.5–97.3)
Specificity, *n*/*N*	44/44	44/44	44/44
%	100	100	100
(95% CI)	(92.0–100)	(92.0–100)	(92.0–100)
ROC area	0.98	0.95	0.97
(95% CI)	(0.97–1.00)	(0.91–0.99)	(0.96–0.99)
Positive predictive value, % (95% CI)*	100 (56.0–100)	100 (54.1–100)	100 (55.5–100)
Negative predictive value, % (95% CI)*	99.2 (98.0–99.6)	97.7 (95.1–98.8)	98.8 (97.7–99.3)

*n* number of accurate test outcomes, *N* number of samples tested, *CI* confidence interval, *ROC* receiver operating characteristics.

*Positive and negative predictive values were calculated with estimated background prevalence of 18.8% (95% CI 16.7–21.1%).

^†^No statistically significant differences between pre-treatment and post-treatment.

### Validation cohort demographics and clinical status

The validation phase prospectively recruited a further 247 febrile patients; 203 (82%) were confirmed *P. knowlesi* malaria cases and 44 were malaria-negative controls. All patients had their malaria status confirmed via reference PCR (Table 1). The median age of patients with *P. knowlesi* malaria was 36 (range 4–87) years, similar to the febrile controls who had a median age of 40 (range 12–80) years. The majority of malaria patients and controls were male, 80% and 70%, respectively. A statistically significant age difference was observed between the sex of the malaria patients, with a median age of 33 (IQR 22–48) years for males and 44 (IQR 29–56) years for females (p = 0.018).

The median duration of self-reported fever prior to presentation was 4 days (range 1–14 days and 2–21 days) for both malaria cases and negative controls respectively. Eighty-three malaria patients (41%) and 5 febrile controls (11%) had a self-reported history of malaria but none within the 2 months prior to study enrolment. The geometric mean parasitaemia of the 203 *P. knowlesi*-infected patients was 837/µL (95% CI 638–1099/µL), ranging between 18 and 331,727/µL. There were 2 adults enrolled with severe malaria according to WHO criteria^22^: one patient had jaundice with a parasite count of 30,906/μL, the other had both jaundice and hyperparasitaemia with a parasite count of 331,727/μL. All other patients were admitted with uncomplicated malaria.

Of the *P. knowlesi*-positive samples tested on the Gazelle™, 143 were collected from patients prior to anti-malarial treatment. There were 60 patients with blood samples collected after administration of anti-malarial treatment, from which 95% (57/60) received at least one dose of oral artemether-lumefantrine and another 5% (3/60) received intravenous artesunate.

### Primary analysis of Gazelle™ diagnostic performance for *P. knowlesi*

From the 143 *P. knowlesi* samples collected and tested before any anti-malarial treatment, the reported test sensitivity was 96.5% (138/143, 95% CI 92.0–98.9%) (Table 2). The geometric mean parasitaemia of these samples was 986/µL (95% CI 719–1352/µL), with the lowest detected parasite count of 18/µL. There were five *P. knowlesi* isolates with false-negative results on Gazelle™ testing, with a median parasitaemia of 42/µL (IQR 41–59, range 23–251/µL). The Gazelle™ recorded a sensitivity of 92.0% (95% CI 84.3–96.7%) for the 81 *P. knowlesi* patients with no previous self-reported malaria history.

There were 60 additional *P. knowlesi*-infected patient samples evaluated after the initial administration of weight-based antimalarial treatment. The median time between treatment and blood sampling for Gazelle™ testing was 2.6 h (IQR 1.5–4.2 h). The geometric mean parasitaemia of samples tested in this group was slightly lower than those tested prior to treatment at 567/µL (95% CI 334–963/µL). When comparing test performance in this group to pre-treatment samples, a trend towards lower test sensitivity of 90.0% (54/60, 95% CI 79.5–96.2%) was observed (p = 0.062). The median parasitaemia of six post-treatment *P. knowlesi* clinical samples with a false-negative result was 40 parasites/µL (IQR 24–52, range 20–244 parasites/µL), with no statistically significant difference in the distribution of parasite counts between pre-and post-treatment groups.

The combined sensitivity for *P. knowlesi* detection including both pre-treatment and post-treatment samples was 94.6% (192/203, 95% CI 90.5–97.3%). The improvement in sensitivity for the validation cohort after refinement of the detection algorithm was not statistically significant compared to the overall pilot phase sensitivity of 92.5%.

The specificity of the Gazelle™ was 100% (95% CI 92.0–100%) for malaria diagnosis, as demonstrated by negative test results for all malaria-negative febrile controls. A high diagnostic accuracy was observed with a ROC of 0.97 (95% CI 0.96–0.99), a positive predictive value (PPV) of 100% (95% CI 55.0–100%) and a negative predictive value (NPV) of 98.8% (95% CI 97.7–99.3%).

### Sensitivity at low parasite counts

**Figure 2:**
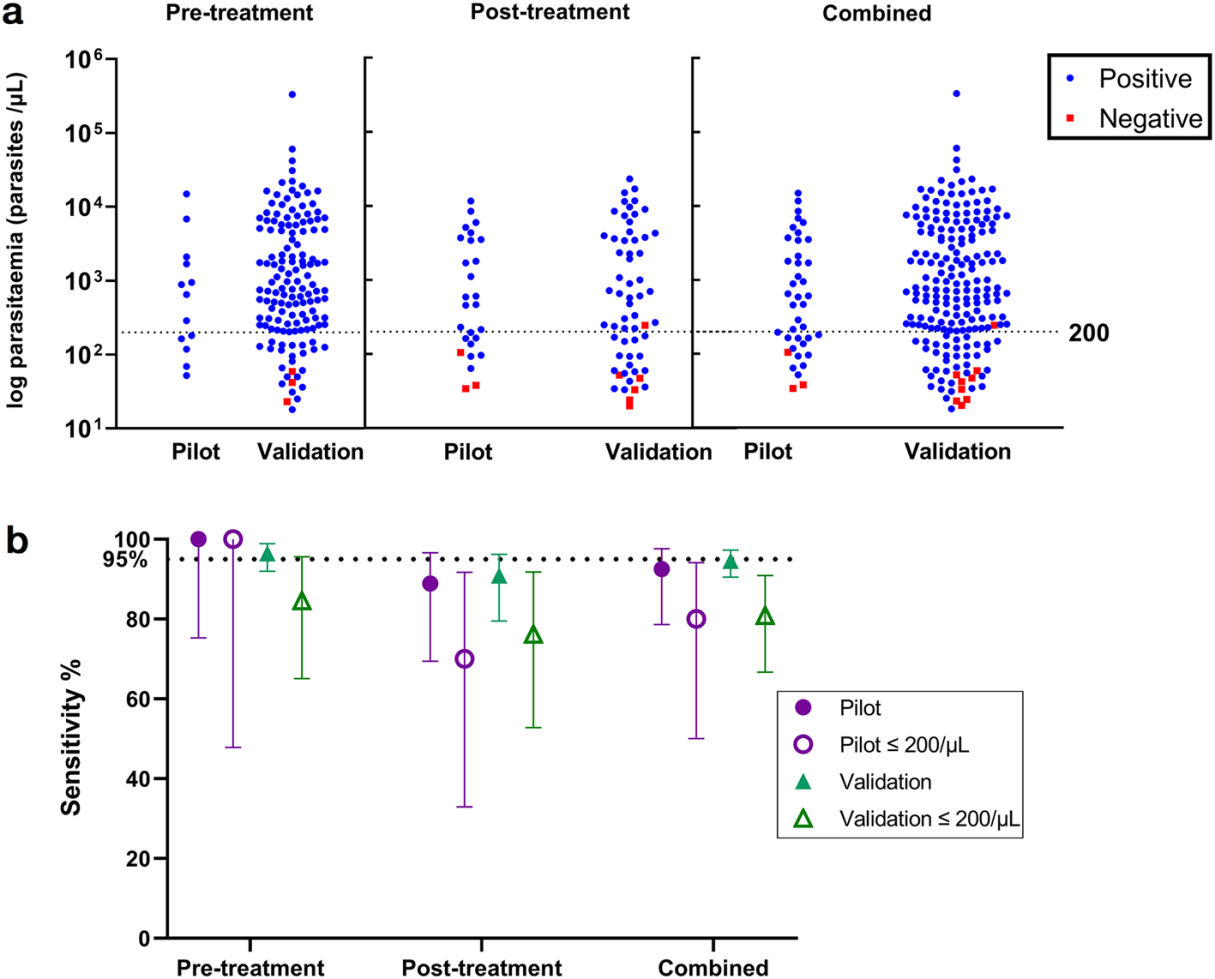
(**a**) Parasite count distribution of *P. knowlesi* clinical samples and corresponding test outcomes, grouped according to antimalarial treatment status at sampling point, further sub-grouped by pilot and validation testing phases; horizontal dotted line denotes 200 parasites/µL cutoff. (**b**) Sensitivity of the Gazelle device accounting for all clinical samples and subset of samples with parasite counts less than 200 parasites/µL, grouped according to antimalarial treatment status at sampling point, further sub-grouped by pilot and validation testing phases; vertical bars represent 95% confidence intervals.

Combining both pilot and validation phase cohorts, 47 *P. knowlesi*-infected patients (23%) had parasite counts less than 200/µL, with a median parasitaemia of 60 (IQR 40–120) parasites/µL. The Gazelle™ achieved a diagnostic sensitivity of 80.9% (95% CI 66.7–90.9%) in this group (Fig. 2). Sensitivity improved to 87.1% (95% CI 78.0–93.3%) for the 85 (42%) *P. knowlesi* malaria cases with a parasitaemia below 500 parasites/µL.

### Sub-microscopic *P. knowlesi samples*

Four patients were censored from the primary analysis for not meeting study inclusion criteria despite initially enrolled as microscopy-negative febrile controls, as subsequent reference PCR confirmed submicroscopic *P. knowlesi* infections. The Gazelle™ did not successfully detect any of these 4 infections, which were below the research microscopy limit of detection of approximately 30 parasites/µL. The inclusion of these submicroscopic *P. knowlesi* infections in the primary analysis would not have resulted in a statistically significant difference in the reported overall Gazelle™ sensitivity.

### Sensitivity of Gazelle™ compared to microscopy

Against reference PCR results, sensitivity of the Gazelle™ (96.5%) was comparable to that of microscopy (97.2%) for *P. knowlesi* detection on samples collected prior to treatment, including the 4 febrile patients with submicroscopic parasitaemia. When compared against all 207 PCR-positive *P. knowlesi* infections before and after treatment, sensitivity of microscopy was higher than the Gazelle™ (95.7% vs 92.8% respectively, p = 0.014).

### Parasite life-stages and Gazelle detection

Microscopic evaluation of parasite life-stages was carried out on the 147 pre-treatment *P. knowlesi* samples. The mean proportions of early trophozoite (rings), late trophozoite and schizont asexual life-stages in a single infection were 2.2% (95% CI 1.4–3.4%), 92.9% (95% CI 90.5–95.2%) and 5.1% (95% CI 3.3–7.8%), respectively. The mean proportion of rings was 1.5% in those positive by the Gazelle™ compared to 6.6% for those that were negative, with no statistically significant difference between groups. Gazelle™ test positivity correlated with parasitaemia (Spearman’s rho = 0.272, p = 0.001), however it did not correlate with the proportion of ring, trophozoite or schizont life-stages within individual *P. knowlesi* infections.

### Detection limits of the Gazelle™ for clinical *P. knowlesi* infections

**Figure 3:**
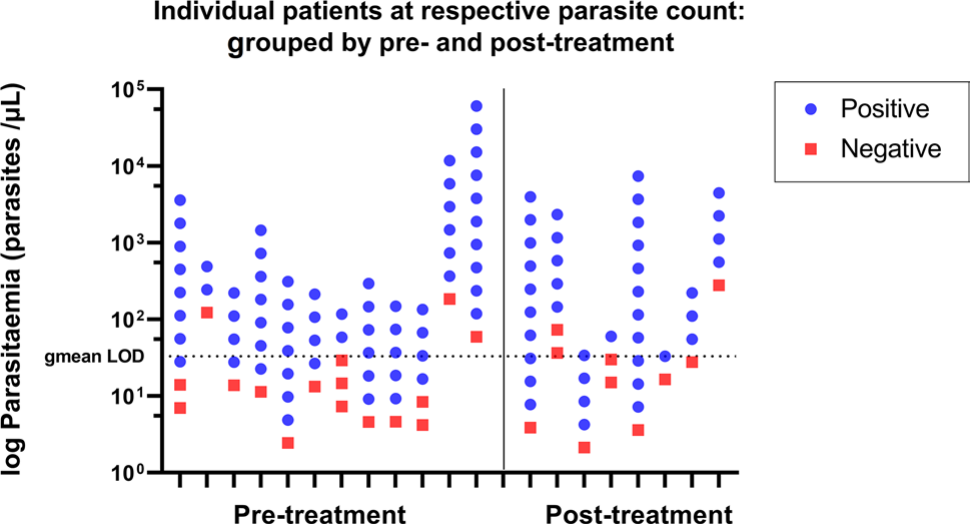
Limit of detection (LOD) of the Gazelle device using individual patient’s parasite counts, grouped according to antimalarial treatment status at sampling point. Horizontal dotted line denotes LOD geometric mean (gmean) was calculated to be 33 parasites/µL.

A subset of 20 *P. knowlesi*-positive patient samples was included in the LOD analysis with serial parasitaemia dilutions. Of the 20 clinical samples, 8 (40%) were obtained from patients who had anti-malarial treatment prior to blood sampling (median time post-treatment 2.7 h, range 0.3–3.7 h). The LOD geometric mean was calculated to be 33 parasites/µL (95% CI 16–65 parasites/µL) (Fig. 3). The individual LOD for the 20 *P. knowlesi* samples evaluated ranged from 4 to 558 parasites/µL. The LOD geometric mean was comparable for pre- versus post-treatment samples: 34 vs 32 parasites/µL, respectively.

## Discussion

Highly sensitive, rapid, and affordable diagnostic tools are urgently needed to detect and improve the clinical management of *P. knowlesi* infections in endemic regions^30^. Sensitive PCR-based assays for detection of *P. knowlesi* have enabled a greater understanding of the heterogenous distribution and increasing incidence of *P. knowlesi* in Malaysia^6^. However, in other endemic areas of Southeast Asia, accurate PCR detection has not been utilised outside targeted malaria research surveys or in returned travelers^31^. The widespread deployment and clinical utility of PCR remain constrained due to availability, cost, and delay in obtaining results. Low density infections for both zoonotic and human-only *Plasmodium* species continue to challenge diagnostic accuracy and hamper national malaria case reporting and elimination efforts^32^. In the current study, the haemozoin-based malaria diagnostic Gazelle™ device demonstrated excellent performance in POC detection of *P. knowlesi*, with a sensitivity of 96.5% for samples tested prior to antimalarial treatment. The high reported sensitivity of the Gazelle™ was above the 95% threshold deemed sufficient by the WHO as an alternative to microscopy for first-line diagnostic use^33^, confirming potential utility of this device in endemic regions where first-line microscopy may not be available. Additionally, a sensitivity of 96% was observed for detection of *P. knowlesi* infections with parasite counts of more than 200/µL, which was previously reported in over 88% of patients passively presenting to health facilities with symptomatic *P. knowlesi* infections^24^.

Previous studies evaluating the clinical performance of the Gazelle™ for malaria diagnosis (*P. falciparum, P. vivax, P. malariae* and *P. ovale spp*) reported sensitivities ranging from 72 to 82% compared to PCR, out-performing current lateral flow malaria RDTs^17,27,34^. The utility of the Gazelle™ as a diagnostic screening tool previously demonstrated 100% specificity for malaria from a survey of 440 febrile individuals in Sri Lanka, although in comparison to light microscopy was unable to differentiate between the small number of *P. falciparum*, *P. ovale*, *P. vivax* and *P. malariae* cases included in the study^34^. Improved sensitivity of the Gazelle™ for *P. vivax* and *P. falciparum* detection has also been reported in patients with no prior history of malaria or treatment^17^, potentially indicating a greater or more rapid degree of haemozoin phagocytosis in individuals with previous malaria exposure. However, this phenomenon was not observed in the current evaluation for *P. knowlesi,* although the small numbers of those with previous *P. knowlesi* infections limited this analysis.

The sensitivity of the Gazelle™ of 81% for *P. knowlesi* infections with parasite counts of less than 200/µL is insufficient for reliable diagnosis in this group, limiting use of the Gazelle™ as an alternative first-line diagnostic tool in areas where microscopy is currently deployed. The performance of the Gazelle™ at low parasite counts was variable overall, highlighted by the lowest recorded parasite count detected at 18/µL, and a mean limit of detection of 33 parasites/µL, similar to microscopy thresholds despite poorer comparative sensitivity against PCR overall. The inconsistent Gazelle™ performance at very low parasite densities likely relates to both inherent variability in the reference standard of microscopic quantification, and also the relative composition of parasite life-stages present in individual infections. A higher proportion of mature trophozoite and schizont life-stages should correspond to a comparatively larger amount of detectable haemozoin in peripheral blood at similar parasite densities. Dark field microscopy image processing algorithms have demonstrated that ring stages older than 6 h begin to show detectable haemozoin, and rings between 10 and 16 h reliably contain detectable haemozoin^16^, implying hemozoin-based detection tools may have limited utility for detection of early infections. Previous evaluations of two other haemozoin-based tools have claimed detection of ring-stage infections of *P. falciparum* is possible at below 40 parasites/µL^20,35^. Despite *P. falciparum* infections intrinsically consisting of only ring-stage parasites in peripheral blood due to cytoadherence of later life-stages in the microvasculature, a separate study assessing the Gazelle™ for clinical *P. falciparum* infections reported false negative at parasite counts up to 1188/µL^34^, with follow-up investigations to rectify this still ongoing.

Recent improvements in lateral flow point-of-care diagnostics for *P. knowlesi* have been based on either non-specific *Plasmodium* species parasite lactate dehydrogenase (pLDH) targets, or cross-reactivity with epitopes for *P. vivax*-specific pLDH^36,37^. However, adapting technology for *P. knowlesi* detection designed for more common human malaria species requires trade-offs between test accuracy, complexity, and cost. The Gazelle™ was tested on a similar parasite density distribution sample set of clinical *P. knowlesi* infections as a previously published large evaluation of 10 pLDH-based malaria rapid diagnostic tests^37^. The best performing *Plasmodium* species pan-pLDH and *P. vivax*-pLDH tests for *P. knowlesi* detection reported sensitivities of 87.0% and 92.0%, respectively^37^, comparable to the overall Gazelle™ performance in the current study. Other antigen- and antibody-based novel diagnostic assays are being designed to detect multiple infectious causes of acute febrile illness, including the DPP® Fever Panel II Asia for diagnosis of falciparum and non-falciparum malaria, dengue and melioidosis^38^. Automated microscopy technology utilising machine learning software to visualize malaria blood films is being developed to detect *Plasmodium* species^39^. Although these devices demonstrate promising alternatives to conventional first-line malaria diagnostics, they have not been evaluated for clinical *P. knowlesi* infections to date. They would ideally require validation to either encompass *P. knowlesi* detection or confirm initial reported specificity for other *Plasmodium* species before field deployment^31^.

A limitation of this study was that the background prevalence of malaria could only be a crude estimation based on patients who presented directly at the referral hospital site. Despite this, a prevalence estimate that is lower by 5% would not significantly affect the NPV (99.1% [95% CI 98.5–99.6%]).

The current Gazelle™ version evaluated in this study provides qualitative malaria diagnosis without the current ability for *Plasmodium* species differentiation and quantification. This study provides important additional data on the first systematic evaluation of a haemozoin-based detection tool for detection of one zoonotic malaria species in an endemic clinical setting. The Gazelle™ device is not able to differentiate between *Plasmodium* species, which is crucial for targeted management in areas where multiple endemic *Plasmodium* species are present. In its current form, the Gazelle™ only allows for rapid screening to differentiate malaria due to any *Plasmodium* species infection from other causes of acute febrile illness for those presenting to health facilities with laboratory capacity, as demonstrated by the consistent 100% specificity from febrile malaria-negative controls observed in this study and other previous evaluations^18,34,40^. Improvements to the Gazelle™ are ongoing, with subsequent versions expected to distinguish between clinically important *Plasmodium* spp*.* via algorithms developed based on species-specific differences in haemozoin morphology^41^.

In addition to detection of malaria based on paramagnetic properties of hemozoin crystals, the current iteration of the Gazelle™ has a separate slot for identifying common haemoglobin variants^42^ using cellulose acetate-based cartridges on a microchip electrophoresis platform. This device has been evaluated for point-of-care screening for other blood-related diseases and hemoglobinopathies such as sickle cell^43,44^ and thalassemia^44^ traits. In malaria endemic areas with a high prevalence of thalassaemia, a single strategically placed device may be able to perform routine screening of thalassemia in addition to rapid detection of clinical malaria infections, thus maximizing its cost-effectiveness in the field.

In terms of diagnostic limitations, there remains the possibility that haemozoin detection methods may give false positive results in cases where circulating haemozoin persist despite the complete clearance of parasites from a previously resolved infection. Murine models have demonstrated the persistent presence of haemozoin for an extended period of 196–270 days post-*Plasmodium* species infection in many organs, including the liver, spleen and bone marrow^45,46^. Haemozoin isolated from cultured *P. falciparum* isolates showed evidence of extreme resistance to phagocytic degradation and was not easily digested by a single process. This suggests haemozoin may be actively redistributed in the body for long periods of time through repeated phagocytosis, not only at the time of liberation from erythrocytes^47^. To our knowledge, the duration by which haemozoin crystals persist in successfully treated *P. knowlesi* infected patients has not been reported. The potential persistence of haemozoin in *P. knowlesi*-infected humans may include retention within the circulatory system or intravascularly in extracellular fluids. It remains unclear what possible immune-modulatory effects they illicit over multiple rounds of phagocytosis, or if the forms in which they persist may be detectable by currently available tools such as the Gazelle™. Despite this theoretical possibility, the current study did show that the Gazelle™ gave 100% specificity for *P. knowlesi* mono-infections; this high specificity may not be achievable in settings of high malaria transmission.

The Gazelle™ offers several major advantages in point-of-care functional utility for malaria diagnosis. This includes minimal time-to-detection of about 1–2 min for the actual blood analysis test component on all study participants. Peripheral blood samples are easily obtainable, particularly given the low blood volume required for testing. This is possible through more acceptable and better tolerated finger-prick sampling of patients compared to more invasive venepuncture. Improving end-user utility would be aided by future designs allowing, i.e., self-loading of 30 µL whole blood directly from finger pricks into cartridge containing the buffer solution, as opposed to requiring pipettes in a laboratory setting. The device is potentially suitable for remote primary health care settings where power supply and laboratory consumable cold chain processes may not be available. In addition, the Gazelle™ requires no pre-requisite manual sample preparation such as Giemsa-staining which may lower overall sensitivity and specificity of test^48^. The Gazelle™ device also provides a comparably quick result compared to that of a typical RDT, which takes about 15 min after loading the blood sample. An economic limitation would be the cost involved in supplying devices to many small clinics. The optimal utility of the Gazelle™ for most low-income countries would be limited to district referral hospitals frequented by many febrile patients daily.

## Conclusion

The Gazelle™ shows potential for use as a non-species-specific tool for malaria diagnosis encompassing *P. knowlesi* detection. The most relevant context would be in countries where other human *Plasmodium* species are approaching elimination and high-quality microscopy is not used as the first-line diagnostic tool. *P. knowlesi* parasitaemia limit of detection of the Gazelle™ was comparable to expert microscopy. Further development of the Gazelle™ is required to accurately differentiate between *Plasmodium* species, including validation for other zoonotic malaria, and to guide appropriate treatment.

## Supplementary Information

Supplementary Information.

## Data Availability

All data generated or analysed during this study are included in this published article and its Supplementary Information file.
